# Aqueous Extract of *Dacryodes edulis* (Burseraceae) Leaves Inhibited Tumor Growth in Female Wistar Rats with 7,12-Dimethylbenz[a]anthracene-Induced Breast Cancer

**DOI:** 10.1155/2021/9960950

**Published:** 2021-07-29

**Authors:** Marie Alfrede Mvondo, Marius Trésor Wego Kamgaing, Sylvie Léa Wansi Ngnokam

**Affiliations:** Research Unit of Animal Physiology and Phytopharmacology, Department of Animal Biology, Faculty of Science, University of Dschang, P. O. Box 67, Dschang, Cameroon

## Abstract

Breast cancer is the most common estrogen-dependent cancer in the world. Hormone therapy for this cancer can be neoadjuvant and/or adjuvant. Herbal remedies with antiproliferative properties are believed to be potential anticancer agents. The aqueous extract of *Dacryodes edulis* (Burseraceae) leaves (AE), a medicinal plant used against cancer in Cameroon, was found to display antiproliferative effects in ovariectomized rats. Compounds isolated from this plant exhibited anticancer activity *in vitro*. To determine whether AE has an anticancer potential, its effects were investigated in rats with already developed breast cancer. Mammary tumors were induced by a single subcutaneous administration (under the mammary gland) of 7,12-dimethylbenz[a]anthracene (DMBA; 50 mg/kgBW) to immature female rats. After 22–26 weeks of observation, animals with palpable tumors were treated with tamoxifen (3.3 mg/kgBW) and AE at doses of 25 and 100 mg/kgBW. The negative control received distilled water. Treatments were given orally for 21 consecutive days. The volume of mammary tumors was evaluated weekly using a caliper. On day 22, animals were sacrificed. Cholesterol and estradiol levels were assessed in serum, breast tumors, mammary glands, and ovaries. Oxidative status of tumors was evaluated. The histological analysis of mammary glands and breast tumors was performed. Results showed that AE reduced tumor volume and weight (*p* < 0.05). This effect was associated with reduced cholesterol (*p* < 0.001) and estradiol (*p* < 0.01) levels in breast tumors, serum, ovaries, and mammary glands. AE also increased tumors levels of malondialdehyde (*p* < 0.05) and antioxidant enzymes (*p* < 0.01). These effects contributed to the decrease in the size of breast alveoli (*p* < 0.01), the density of cancer cells in breast tumors, and the invasion of these cells into the tumor connective tissue. In conclusion, the aqueous extract of *D. edulis* leaves, thanks to its ability to inhibit tumor growth, could be considered as a potential alternative for the neoadjuvant treatment of estrogen-dependent breast cancer.

## 1. Introduction

Cell proliferation is the process strongly involved in cancer genesis [1]. This latter represents the main cause of death all over the world, with 19.3 million cases and 10 million deaths in 2020 [2]. In this proportion, estrogen-dependent cancers were reported to be the most common cancers (50–80%) [3]. These types of cancers are made up of cells that express estrogen receptors and whose proliferation is stimulated by estrogens [4]. Estrogens are steroid hormones capable of promoting cell cancers growth and development [4], especially in estrogen-sensitive organs such as breast tissue. The literature reports that breast cancer is the most prevalent estrogen-dependent cancer in women in developing countries [5, 6]. In line with this, these malignant diseases globally represent 23% of women cancers and 11.7% of all cancers [2]. According to Torre et al. [7], breast and cervical cancers are responsible for the high female mortality rate (around 45.4%) recorded in the countries of Sub-Saharan Africa in 2012. In Cameroon, breast cancer was found to be the most common cancer among women in 2012 with an age standardized ratio (ASR) of 2,625 new cases per 100,000 women per year [8] and an incidence of 25.89% [9]. Two years later, this incidence increased to 34% [10]. A recent study reports that the incidence of breast cancer in 2020 was estimated at 34.1% in Cameroon [11]. Moreover, the majority of these breast cancers are detected at an advanced stage, resulting in a low survival rate. Indeed, according to Nguefack et al. [12] and Ngowa et al. [13], breast cancers screening was found to be elevated at stages III and IV of the disease in Cameroon, leading to poor treatment outcomes [12, 13].

The clinical management of breast cancer involves the use of selective estrogen receptor modulators like tamoxifen and fulvestrant (for hormonal therapies), aromatase inhibitors like anastrozole (Arimidex), exemestane (Aromasin), and letrozole (Femara), CAR-T therapy, cytotoxic antibiotics such as atezolizumab (Tecentriq) and sacituzumab govitecan-hziy (Trodelvy), and, in certain conditions, the surgical removal of tumors such as lumpectomy (sentinel node biopsy) or partial mastectomy associated with radiation therapy to minimize recurrences [14–16]. However, these therapies remain unsatisfactory because of the high cost of treatments, their side effects, recurrences, and resistance of cancer cells to treatment. Tamoxifen, for example, is the most common hormone therapy used in the management of estrogen-sensitive (ER+) breast cancer. The long-term use of this hormone therapy was reported to induce uterine endometrial hyperplasia, increasing the risks of endometrial cancer [17, 18], hence the need to find effective, affordable, and better tolerated alternatives. Previous scientific works have reported the anticancer effects of several Cameroonian medicinal plants. These include *Ficus umbellata* [19], *Acacia seyal* [20], *Azadirachta indica* [21], and *Anthonotha macrophylla* [22, 23]. Several of these plants were found to induce antiproliferative effects *in vitro* and *in vivo*. These studies were performed on a preventive regimen in order to manage breast cancer at an early stage. However, one of the main problems with breast cancer in Cameroon is that it is usually diagnosed at an advanced stage, resulting in poor treatment outcomes [12, 13]. Numbers of patients therefore generally turn to herbal medicine with already advanced breast cancer, either because of the late diagnosis or because of recurrences and resistance to conventional anticancer treatments. The search for alternative treatments adapted to subjects suffering from advanced breast cancer is therefore topical.

*Dacryodes edulis* (G. Don) H. J Lam (Burseraceae) is a medicinal plant traditionally used in Africa to cicatrize wound [24] and to treat diverse ailments including skin diseases and inflammation [24–26]. Scientific investigations reported a significant antioxidant capacity of *D. edulis* [27]. This antioxidant capacity of *D. edulis* led some authors to suggest that this plant may help in preventing oxidative damage associated with cancer [28]. A phytochemical assay we recently performed [4] with the aqueous extract of *D. edulis* leaves indicated the presence of a wide range of bioactive compounds (flavonoids, phenols, alkaloids, and flavonols) endowed with antiproliferative and anticancer activities [29]. In addition, this plant extract was reported to inhibit cell growth and proliferation induced by estradiol on the uterus and vagina of female *Wistar* rats [4], supporting in part the hypothesis according to which this plant may help against cancer diseases [28]. In order to provide more scientific evidence to the probable anticancer potential of *D. edulis*, we propose to evaluate the effects of the aqueous extract of *D. edulis* leaves in animals with an already developed breast cancer. The effects of the aforementioned extract were investigated on tumor growth, the oxidative status of breast tumors, and the histomorphology of breast tumors and mammary glands. The determination of serum and tissue (breast tumors, mammary glands, and ovaries) levels of total cholesterol and estradiol allowed elucidating the probable mechanism of action of this plant.

## 2. Materials and Methods

### 2.1. Chemicals

7,12-dimethylbenz[a]anthracene (DMBA) was obtained from Sigma Aldrich, D3254-1G, Lot# SLBS1630V (St. Louis, USA). Tamoxifen citrate was provided by EuroGenerics (EG) Lab, Egis Pharmaceuticals Ltd, 1106 Budapest, Hungary. Total cholesterol levels were evaluated using diagnostic kits obtained from Sigma-Aldrich (Stanford, Germany). Mouse/Rat Estradiol ELISA kit was purchased from Calbiotech (El Cajon, California, USA).

### 2.2. Plant Collection, Authentication, and Extraction

#### 2.2.1. Plant Collection and Authentication

The leaves, stem barks, and fruits of *D. edulis* were collected in Dschang (West region of Cameroon) in August 2018. They were compared to the botanical sample of Letouzez R. N. 4685 registered at the Cameroon National Herbarium and the plant was authenticated under the number 5552 SRF/CAM (YA) [4].

#### 2.2.2. Plant Extraction

The aqueous extract of *D. edulis* leaves was prepared following the method we have previously described [4, 30, 31]. Briefly, fresh and clean leaves of *D. edulis* were air-dried in the shade and ground. 300 g of the resulting powder was macerated in 3 L of distilled water for 48 h at room temperature. The supernatant was filtered using Whatman paper number 4 and dried in an oven at 40°C for two days. This process allowed obtaining 29 g (yield: 9.67%) of the aqueous extract. The aforesaid extract was kept in an air-tight container at −20°C until use.

#### 2.2.3. Dose Determination

To evaluate the anticancer properties of the aqueous extract of *D. edulis* leaves, two doses (25 and 100 mg/kgBW) were administered to animals. These doses come from our previous study reporting their ability to inhibit cell growth and proliferation in primary estrogen target tissues (uterus and vagina) of ovariectomized female *Wistar* rats [4]. The same doses of this extract were used in this study to find out whether this extract could be effective against breast cancer, another estrogen-sensitive tissue [32].

#### 2.2.4. Experimental Animals

Immature female *Wistar* rats (35–45 days old) weighing 45–50 g were provided from the breeding facility of the Research Unit of Animal Physiology and Phytopharmacology, University of Dschang (Cameroon). They were bred and kept under a standard soy-free rat diet in order to reduce exposure to exogenous estrogenic compounds [4]. Animals had free access to diet and water *ad libitum*.

#### 2.2.5. Ethical Approval

Animal handling and *in vivo* experiments were carried out after the approval of the research proposal by the scientific committee of the Department of Animal Biology, University of Dschang, on 15 May 2018, in conformity with the European community guidelines EEC Council Directive 2010/63/EU of 22 September 2010 [33].

#### 2.2.6. Study Design

The anticancer effect of the aqueous extract of *D. edulis* leaves on DMBA-induced mammary tumors was investigated following the method described by Zingue et al. [20] with slight modifications. Briefly, 67 immature female *Wistar* rats aged 35–45 days were used. 62 of them received under anesthesia (10 mg/kgBW diazepam and 50 mg/kgBW ketamine, intraperitoneally) a single dose (50 mg/kgBW) of DMBA subcutaneously under the mammary gland. The 5 remaining rats served as the normal control. Animals which received DMBA were followed up and breasts palpated once a week until the development of tumors. Breast tumors were palpable 22–26 weeks after the subcutaneous administration of a single dose of DMBA. After measuring the dimensions of tumors (length, width, and height) using a caliper, the volume of tumors was determined using the ellipsoid volume formula:

(1) $$\begin{array}{c} \frac{\pi }{6}\times \text{length}\times \text{width}\times \text{height.} \end{array}$$

Animals with palpable tumors (*n* = 25) were then assigned to the following treatment groups (*n* = 5):

- Normal control: healthy animals receiving distilled water, per os
- DMBA + DW: animals with cancer receiving distilled water, per os
- DMBA + TAM: animals with cancer treated with tamoxifen (3.3 mg/kgBW), per os
- DMBA + AE 25: animals with cancer treated with the aqueous extract of *D. edulis* leaves at the dose of 25 mg/kgBW, per os
- DMBA + AE 100: animals with cancer treated with the aqueous extract of *D. edulis* leaves at the dose of 100 mg/kgBW, per os

Tamoxifen was used as reference substance in this study because hormone therapy of breast cancer can be administered either before surgery (neoadjuvant treatment) to reduce the size of a large tumor and/or after surgery (adjuvant treatment) to destroy the cancer cells that remain after surgery and radiotherapy, thus preventing recurrences [34, 35]. Treatments were administered by gavage for 21 consecutive days. Tumor volume was assessed once a week during the entire treatment period. At the end of the 21-day treatment, animals were submitted to a 12 h overnight nonhydric fasting and were anesthetized thereafter using an intraperitoneal injection of diazepam (10 mg/kgBW) and ketamine (50 mg/kgBW). Following anesthesia, animals were sacrificed by incision of the abdominal artery. Blood was collected from each rat in dry tubes (without an anticoagulant) using a catheter inserted into the aforementioned artery. The tubes were kept at room temperature and serum was separated by centrifugation at 3000 rpm for 15 min. The serum obtained was kept at −20°C until use. Breast tumors were collected, cleaned of fat, and weighed. Each tumor was then divided into two sections. One section was homogenated in 0.9% NaCl (0.1 g per 1 ml) and centrifuged at 3000 rpm for 15 min. The resulting supernatant was kept at −20°C until use. The other section was fixed in 10% formalin for histological analysis. A sample of the mammary gland and the ovaries was collected, homogenated in 0.9% NaCl (0.1 g per 1 ml), and centrifuged at 3000 rpm for 15 min. The resulting supernatant was kept at −20°C until use. Another mammary gland was fixed in 10% formalin for histological analysis.

### 2.3. Biochemical Analysis

#### 2.3.1. Evaluation of Total Cholesterol and Estradiol Levels

Total cholesterol levels were evaluated in serum, ovaries, tumors, and mammary glands by fully automated (Cobas Mira S autoanalyzer) enzymatic method using a reagent kit provided by Sigma Diagnostics (Budapest, Hungary) [4].

Levels of estradiol were also assessed in serum, ovaries, tumors, and mammary glands. Estradiol assay was performed using a reagent kit obtained from Calbiotech (El Cajon, California, USA). Enzyme-linked immunosorbent assay (ELISA) test was performed to determine the absorbance of the sample and calibrator using a Multiskan ascent plate reader (ELISA microplate reader, purchased from MTX Lab Systems). The means obtained from the absorbance of the calibrators provided with the kit were used for the establishment of the calibration curve, from which the concentration of estradiol in the sample was assessed [4].

#### 2.3.2. Determination of Oxidative Stress Biomarkers and Total Protein Levels

Malondialdehyde (MDA) levels were evaluated in breast tumors by the method described by Wilbur et al. [36]. This method is based on the formation of a pink pigment with a maximum absorption at 532 nm. The determination of MDA levels was performed using the following formula:

(2) $$\begin{array}{c} \left[\text{MDA}\right]=\frac{\Delta \text{DO}}{\varepsilon \text{.l.m}}, \end{array}$$

where [MDA]  = concentration of MDA (nM/mg of tissue); ΔDO = absorbance of the sample-absorbance of the reagent blank; *ε* = molar extinction coefficient (1.56 × 10–4 nM^−1^cm^−1^); *l* = path length (1 cm); *m* = mass of the tissue collected for homogenization (mg).

The activity of catalase in breast tumors was evaluated using the method described by Sinha [37], based on the estimation of the concentration of undecomposed H_2_O_2_ in water. The activity of catalase in the sample was evaluated using the following formula:

(3) $$\begin{array}{c} \text{C.A}=\frac{\Delta \text{DO}}{\text{a.t.p}}, \end{array}$$

where C.A = catalase activity (*μ*moL of H_2_O_2_/min/g of total proteins); ΔDO = absorbance of the sample-absorbance of the reagent blank; *a* = slope of the calibration curve; *t* = reaction time (1 min); *p* = tumor total protein level (g).

The determination of tumor levels of superoxide dismutase (SOD) was performed by the method described by Misra and Fridovich [38] and Dimo et al. [39]. During this test, the absorbance of the product (adrenochrome) obtained after initiating the reaction was evaluated after 30 and 90 seconds at 480 nm and the inhibition percentage (I) of the SOD was determined as follows:

(4) $$\begin{array}{c} \text{\%I }=\left[100-\left(\frac{\text{absorbance of the sample}}{\text{absorbance of the reagent blank}}\right)\right]\times 100. \end{array}$$

One unit of SOD was equivalent to 50% inhibition and the determination of tissue activity of SOD was performed using the following formula:

(5) $$\begin{array}{c} \text{A }=\;\frac{\text{I}}{50}\times \text{tumor total protein level.} \end{array}$$

The evaluation of total protein levels in breast tumors was performed using a reagent kit purchased from Randox (London, UK), following the manufacturer's instructions.

### 2.4. Histological Analysis

The histological analysis of the tumors and mammary glands was performed on 5 *μ*m sections of paraffin-embedded tissues submitted to a hematoxylin-eosin staining. Microphotographies of tumor tissue and breast lobule were obtained using a computer connected to a light microscope provided by Olympus (Tokyo, Japan). These images were transferred and analyzed with the ImageJ 1.3 software [4]. The average size of mammary glands was obtained by measuring the size of alveoli of each animal in a group and the average value of the group was calculated.

### 2.5. Statistical Analysis

Data were analyzed using the GraphPad Prism software version 5.03. One-way analysis of the variance (ANOVA) followed by the Tukey post hoc test for multiple comparisons was used for the analysis of data with one variable (treatments). ANOVA repeated measures followed by the Bonferroni post hoc test for multiple comparisons were used to analyze data with two variables (time and treatments). Data are presented as mean ± standard error of the mean (SEM). Differences were considered significant at *p* < 0.05.

## 3. Results

### 3.1. Effects of Treatments on the Volume and Weight of Breast Tumors

Following the 21-day treatment, the tumor volume of the DMBA + DW group was set at 3.98 ± 0.79 cm^3^. Tamoxifen reduced this volume to 2.40 ± 0.34 cm^3^ (38% reduction; *p* < 0.05). A similar observation was made with the aqueous extract of *D. edulis* leaves at tested doses (tumor volume: 3.56 ± 0.71 cm^3^ (11% reduction) at 25 mg/kgBW and 3.15 ± 0.63 cm^3^ (21% reduction) at 100 mg/kgBW) (Table 1).

The weight of breast tumors was set at 3.87 ± 0.77 g in the DMBA + DW group. Following treatment with tamoxifen, this parameter decreased to 1.40 ± 0.28 g (63.82% reduction; *p* < 0.001) as compared to the DMBA + DW control group. A similar decrease was observed with the aqueous extract of *D. edulis* leaves at tested doses [tumor weight: 2.44 ± 0.49 g (36.95% decrease; *p* < 0.05) at 25 mg/kgBW and 1.95 ± 0.39 g (49.61% decrease; *p* < 0.001) at 100 mg/kgBW], as compared to the DMBA + DW control group.

**Figure 1:**
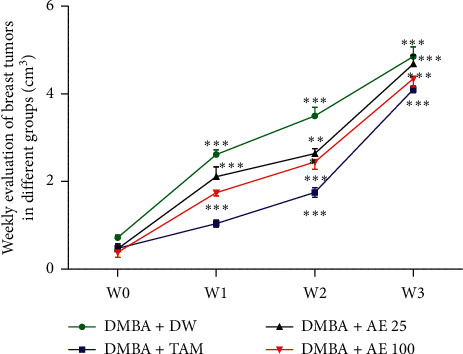
Effects of treatments on tumor growth. W0: week 0; W1: week 1; W2: week 2; W3: week 3; DMBA: 7,12-dimethylbenz[a]anthracene; DW: distilled water; TAM: tamoxifen; AE: aqueous extract of *D. edulis* leaves. Data are expressed as mean ± SEM (*n* = 5); ^*∗∗*^*p* < 0.01 and ^*∗∗∗*^*p* < 0.001 as compared to W0.

Results from Figure 1 show a gradual increase in the volume of breast tumors in the different groups, from week 1 to week 3. In the DMBA + DW control group, this increase was 112% (*p* < 0.001), 1306% (*p* < 0.001), and 2312% (*p* < 0.001) at weeks 1, 2, and 3, respectively, compared to week 0. In the DMBA + TAM group, the volume of breast tumors increased by 80%, 309% (*p* < 0.001), and 545% (*p* < 0.001) at weeks 1, 2, and 3, respectively, as compared to week 0. Following treatment with the aqueous extract of *D. edulis* leaves at the dose of 25 mg/kgBW, this parameter increased by 149% (*p* < 0.001) at week 1, by 741% (*p* < 0.001) at week 2, and by 1254% (*p* < 0.001) at week 3, as compared to week 0. A similar effect was observed at the dose of 100 mg/kgBW of the same extract: 165% (*p* < 0.001) increase at week 1, 499% (*p* < 0.001) increase at week 2, and 636% (*p* < 0.01) increase at week 3, as compared to week 0.

Table 2 shows the percentage inhibition of tumor growth induced by the treatments relative to the DMBA + DW group. It comes out from this table that, before treatments (week 0), the volumes of breast tumors were almost similar in the different groups. After 1, 2, and 3 weeks of treatment, tamoxifen decreased tumor volume by 60% (*p* < 0.001), 50% (*p* < 0.001), and 38% (*p* < 0.05), respectively, in comparison with the DMBA + DW control group. Similar observation was made with the aqueous extract of *D. edulis* leaves at tested doses. Indeed, at the dose of 25 mg/kgBW, the aqueous extract of *D. edulis* leaves decreased the volume of breast tumors by 19%, 25% (*p* < 0.01), and 11%, after 1, 2, and 3 weeks of treatment, respectively, as compared to the DMBA + DW control group. At the dose of 100 mg/kgBW, this parameter decreased by 33% (*p* < 0.01), 42% (*p* < 0.001), and 21% after 1, 2, and 3 weeks of treatment, respectively, as compared to the DMBA + DW control group.

### 3.2. Effects of Treatments on Serum, Ovary, Mammary Gland, and Tumor Levels of Total Cholesterol

**Figure 2:**
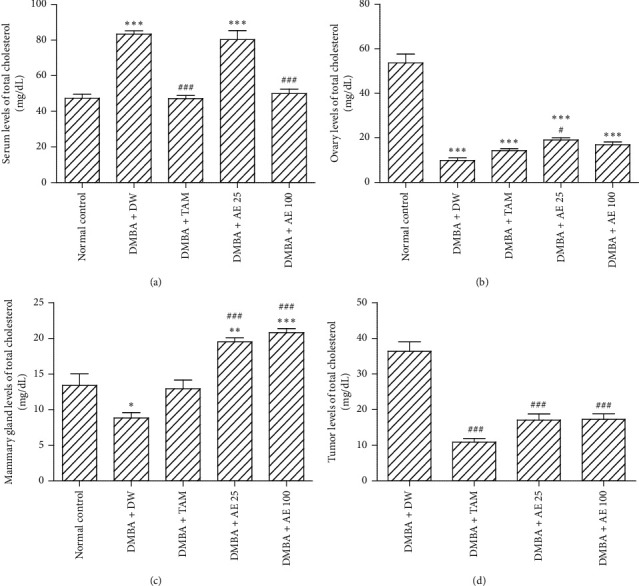
Effects of treatments on serum (a), ovary (b), mammary gland (c), and tumor (d) levels of total cholesterol. DMBA: 7,12-dimethylbenz[a]anthracene; DW: distilled water; TAM: tamoxifen; AE: aqueous extract of *D. edulis* leaves. Data are expressed as mean ± SEM (*n* = 5); ^*∗*^*p* < 0.05, ^*∗∗*^*p* < 0.01, and ^*∗∗∗*^*p* < 0.001 as compared to the normal control; ^#^*p* < 0.05 and ^###^*p* < 0.001 as compared to DMBA + DW.

Compared to the normal control group, serum levels of total cholesterol increased by 76.18% (*p* < 0.001) in the DMBA + DW group (Figure 2(a)). Tamoxifen decreased this parameter by 43.42% (*p* < 0.001), as compared to the DMBA + DW control group. At the dose of 100 mg/kgBW, the aqueous extract of *D. edulis* leaves also reduced (40.15% induction, *p* < 0.001) serum levels of cholesterol, as compared to the DMBA + DW control group.

Ovary levels of total cholesterol decreased by 81.59% (*p* < 0.001) in the DMBA + DW group, as compared to the normal control group (Figure 2(b)). Tamoxifen increased this parameter by 44.67%, as compared to the DMBA + DW control group. The aqueous extract of *D. edulis* leaves further increased the levels of ovary total cholesterol at tested doses: 93.49% induction at the dose of 25 mg/kgBW (*p* < 0.05) and 71.12% induction at the dose of 100 mg/kgBW, as compared to the DMBA + DW control group.

Figure 2(c) shows that the levels of total cholesterol in the mammary gland decreased by 33.98% (*p* < 0.05) in the DMBA + DW group, as compared to the normal control group. Tamoxifen increased this parameter by 46.24%, as compared to the DMBA + DW control group. The aqueous extract of *D. edulis* leaves further increased this parameter at tested doses: 120.73% induction (*p* < 0.001) at the dose of 25 mg/kgBW and 135.14% induction (*p* < 0.001) at the dose of 100 mg/kgBW, as compared to the DMBA + DW control group.

Tumor levels of total cholesterol decreased by 70.1% (*p* < 0.001) in animals treated with tamoxifen, as compared to the DMBA + DW control group. A similar decrease was observed in animals treated with the aqueous extract of *D. edulis* leaves at tested doses: 53% induction (*p* < 0.001) at the dose of 25 mg/kgBW and 52.44% induction (*p* < 0.001) at the dose of 100 mg/kgBW, as compared to the DMBA + DW control group (Figure 2(d)).

### 3.3. Effects of Treatments on Serum, Ovary, Mammary Gland, and Tumor Levels of Estradiol

**Figure 3:**
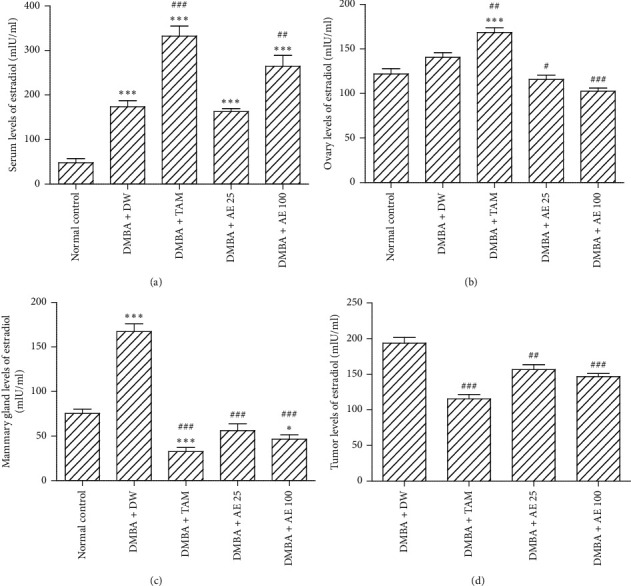
Effects of treatments on serum (a), ovaries (b), mammary gland (c), and tumor (d) levels of estradiol. DMBA: 7,12-dimethylbenz[a]anthracene; DW: distilled water; TAM: tamoxifen; AE: aqueous extract of *D. edulis* leaves. Data are expressed as mean ± SEM (*n* = 5); ^*∗*^*p* < 0.05 and ^*∗∗∗*^*p* < 0.001 as compared to the normal control. ^#^*p* < 0.05, ^##^*p* < 0.05, and ^###^*p* < 0.001 as compared to DMBA + DW.

Serum levels of estradiol increased by 261.51% (*p* < 0.001) in the DMBA + DW group, as compared to the normal control group. Tamoxifen increased this parameter by 91.4% (*p* < 0.001), as compared to the DMBA + DW control group. In comparison with the same control group (DMBA + DW), the aqueous extract of *D. edulis* leaves (100 mg/kgBW) increased serum levels of estradiol by 52.26% (*p* < 0.01) (Figure 3(a)).

Ovary levels of estradiol increased by 15.4% in the DMBA + DW group, as compared to the normal control group. Tamoxifen further increased this parameter by 19.79% (*p* < 0.01), in comparison with the DMBA + DW control group. The aqueous extract of *D. edulis* leaves on the contrary reduced ovary levels of estradiol at tested doses: 17.71% induction at 25 mg/kgBW (*p* < 0.05) and 27% induction at 100 mg/kgBW (*p* < 0.001), in comparison with the DMBA + DW control group (Figure 3(b)).

Figure 3(c) shows that the levels of estradiol in mammary glands increased by 121.96% (*p* < 0.001) in the DMBA + DW group, as compared to the normal control group. Tamoxifen decreased this parameter by 80.59% (*p* < 0.001), as compared to the DMBA + DW control group. Similarly, the aqueous extract of *D. edulis* leaves decreased the levels of estradiol in mammary glands by 66.38% at the dose of 25 mg/kgBW (*p* < 0.001) and by 72.14% at the dose of 100 mg/kgBW (*p* < 0.001), as compared to the DMBA + DW control group.

Tamoxifen decreased tumors levels of estradiol by 40.5% (*p* < 0.001), as compared to the DMBA + DW control group (Figure 3(d)). The aqueous extract of *D. edulis* leaves induced a similar effect as it decreased this parameter by 19.22% (*p* < 0.01) and 24.28% (*p* < 0.001) at doses of 25 and 100 mg/kgBW, respectively, in comparison with the DMBA + DW control group.

### 3.4. Effects of Treatments on Oxidative Stress-Related Parameters in Tumors

Table 3 shows that tamoxifen increased tumors levels of MDA by 35%, as compared to the DMBA + DW control group. The aqueous extract of *D. edulis* leaves further increased this parameter at doses of 25 mg/kgBW (46.2% induction) and 100 mg/kgBW (52.1% induction; *p* < 0.05), as compared to the DMBA + DW control group.

The tumor levels of catalase increased by 161.11% (*p* < 0.001) in animals treated with tamoxifen, as compared to the DMBA + DW control group (Table 3). A similar increase in this parameter was observed in the tumors of animals treated with the aqueous extract of *D. edulis* leaves at doses of 25 mg/kgBW (94.44% induction; *p* < 0.01) and 100 mg/kgBW (133.33% induction; *p* < 0.001), as compared to the DMBA + DW control group.

The activity of SOD increased by 48.3% (*p* < 0.001) in the tumors of animals treated with tamoxifen, in comparison with the DMBA + DW control group. In comparison with the same group (DMBA + DW), the aqueous extract of *D. edulis* leaves induced a similar effect as it increased the activity of SOD at doses of 25 mg/kgBW (19.44% induction) and 100 mg/kgBW (35.1% induction; *p* < 0.01) (Table 3).

**Figure 4:**
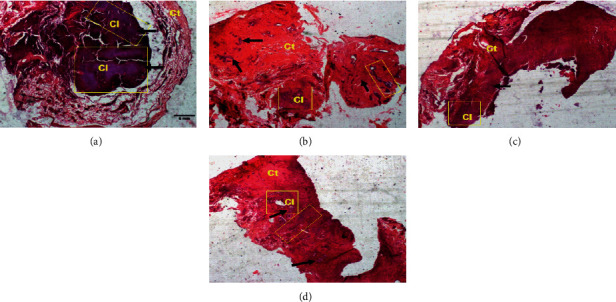
Photomicrographs (hematoxylin-eosin staining, 100x) showing the effects of treatments on the extent of cancerous lesion in breast tumors. Yellow frame: extent of cancerous lesion; Cl: cancerous lesion; Ct: connective tissue; black arrow: cancer cells; DMBA: 7,12-dimethylbenz[a]anthracene; DW: distilled water; TAM: tamoxifen; AE: aqueous extract of *D. edulis* leaves. Data are expressed as mean ± SEM (*n* = 5). (a) DMBA + DW. (b) DMBA + TAM. (c) DMBA + AE 25. (d) DMBA + AE 100.

Figure 4 shows photomicrographs (hematoxylin-eosin staining, ×100) of the 10^th^ sections of the breast tumors. It can be seen from this figure that, in comparison with the DMBA + DW control group, tamoxifen reduced the extent of the cancerous lesion which boils down to a few clusters of cancer cells within the tumors. A similar observation was noticed with the aqueous extract of *D. edulis* leaves at tested doses.

**Figure 5:**
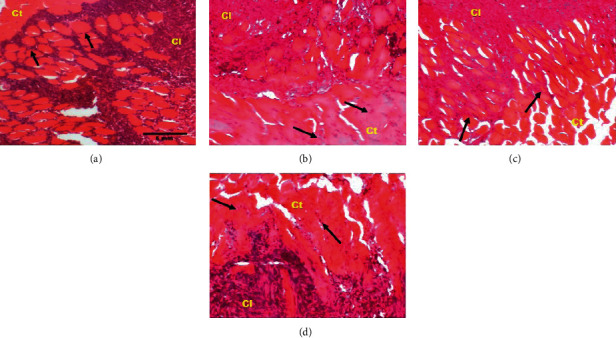
Photomicrographs (hematoxylin-eosin staining, 400x) showing the effects of treatments on the invasion of the tumor connective tissue by cancer cells. Cl: cancer lesion; Ct: connective tissue; black arrow: cancer cells' infiltration; DMBA: 7,12-dimethylbenz[a]anthracene; TAM: tamoxifen; AE: aqueous extract of *D. edulis* leaves. Data are expressed as mean ± SEM (*n* = 5). (a) DMBA + DW. (b) DMBA + TAM. (c) DMBA + AE 25. (d) DMBA + AE 100.

Figure 5 shows a 400x magnification of microphotographs of breast tumors. In the DMBA + DW group, cancer cells massively invaded the connective tissue. Following treatment with tamoxifen (3.3 mg/kgBW), we observed a regression of cancer cells infiltration in the connective tissue. At the dose of 100 mg/kgBW, the aqueous extract of *D. edulis* leaves also reduced the invasion of cancer cells in the connective tissue.

### 3.5. Histomorphology of the Mammary Gland

**Figure 6:**
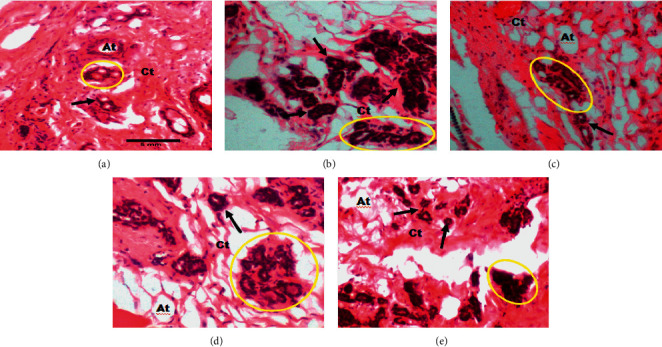
Photomicrographs (hematoxylin-eosin staining, 200x) showing the effects of treatments on the mammary glands. At: adipose tissue; Ct: connective tissue; yellow circle: lobule; black arrow: alveoli; DMBA: 7,12-dimethylbenz[a]anthracene; DW: distilled water; TAM: tamoxifen; AE: aqueous extract of *D. edulis* leaves. Data are expressed as mean ± SEM (*n* = 5). (a) Normal control. (b) DMBA + DW. (c) DMBA + TAM. (d) DMBA + AE 25. (e) DMBA + AE 100.

**Figure 7:**
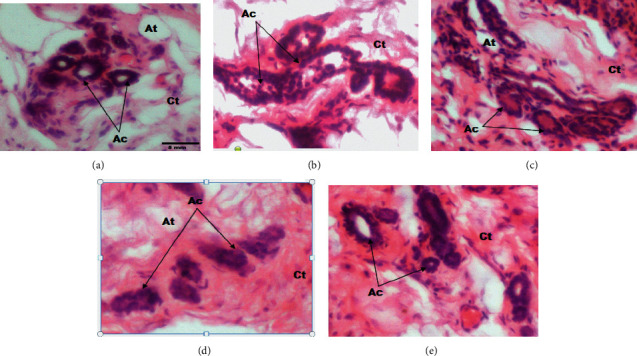
Photomicrographs (hematoxylin-eosin staining, 400x) showing the effects of treatments on the mammary glands. At: adipose tissue; Ct: connective tissue; Ac: alveoli; DMBA: 7,12-dimethylbenz[a]anthracene; DW: distilled water; TAM: tamoxifen; AE: aqueous extract of *D. edulis* leaves. Data are expressed as mean ± SEM (*n* = 5). (a) Normal control. (b) DMBA + DW. (c) DMBA + TAM. (d) DMBA + AE 25. (e) DMBA + AE 100.

Figure 6 shows 200x magnification photomicrographs of the mammary glands. According to this figure, the mammary gland of animals from the DMBA + DW group presented hypertrophic lobules with very large alveoli, compared to the normal control group. Tamoxifen reduced the size of lobules and alveoli. Similar atrophied lobules and alveoli were observed in the mammary gland of animals treated with the aqueous extract of *D. edulis* leaves at the dose of 100 mg/kgBW. These observations are supported by Figure 7, which presents 400× magnification photomicrographs of the mammary glands. This figure clearly shows that the diameter of alveoli in the DMBA + DW group increased significantly as compared to that of the normal control group. Following treatments, this parameter (diameter of alveoli) reduced and was similar to that of the normal control group.

**Figure 8:**
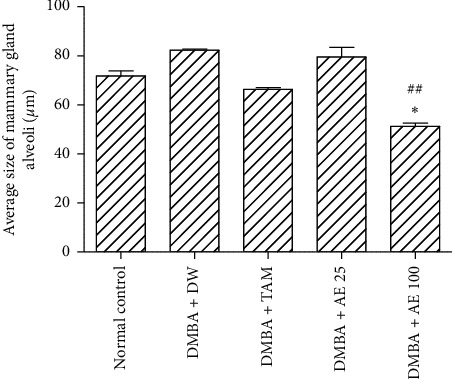
Effects of treatments on the size of mammary gland alveoli. DMBA: 7,12-dimethylbenz[a]anthracene; DW: distilled water; TAM: tamoxifen; AE: aqueous extract of *D. edulis* leaves. Data are expressed as mean ± SEM (*n* = 5). ^*∗*^*p* < 0.05 as compared to the normal control. ^##^*p* < 0.001 as compared to DMBA + DW.

Figure 8 shows that the average length of mammary gland alveoli increased by 11.5% in the DMBA + DW group, as compared to the normal control group. Tamoxifen decreased the size of alveoli by 19%, as compared to the DMBA + DW control group. At the dose of 100 mg/kgBW, the aqueous extract of *D. edulis* leaves decreased this parameter by 39.12% (*p* < 0.01), as compared to the DMBA + DW control group.

## 4. Discussion

The exposition to environmental pollutants such as DMBA induced mutation, DNA damage, oncogene activation, and cancer genesis [40, 41]. The administration of a single dose of DMBA (50–80 mg/kgBW) to female rats was reported to induce breast cancer development [21, 41–44]. In agreement with this report, our results showed that, after the subcutaneous administration of a single dose of DMBA (50 mg/kgBW) under the mammary gland of female *Wista*r rats, breast tumors developed 22–26 weeks later, with an induction percentage of 40%. This result is in accordance with the observation of Alvarado et al. [42] reporting that the intraperitoneal administration of a single dose of DMBA (50 mg/kgBW) to juvenile female rats induced mammary tumor development after more than 20 weeks [42].

The increase of tumors volume observed in the DMBA + DW group was associated with the increase of serum levels of total cholesterol and a decrease of this parameter in the mammary gland. As reported by Maud [45], estradiol is derived from cholesterol and is widely involved in tumor progression. The decrease in mammary gland levels of total cholesterol would therefore reflect the use of this lipid in the process of steroidogenesis [45]. Contrariwise, in serum and breast tumors, increased levels of cholesterol were associated with increased levels of estradiol. This could be the result of an increased production of cholesterol stimulated by DMBA and a sustained process of steroidogenesis. Therefore, in addition to the ability of this environmental pollutant to stimulate steroidogenesis [46], DMBA may also promote the synthesis of the precursor of steroid hormones: cholesterol. The increase of estradiol levels would justify the increased invasion and density of cancer cells in breast tumors and the hypertrophy of breast lobules, taking into account the significant role of estrogens in the stimulation of cell proliferation [4, 44].

In the clinical management of estrogen-dependent breast cancer, hormone therapy is prescribed as neoadjuvant treatment and/or adjuvant treatment to decrease the size of a large tumor before surgery and to prevent recurrences after surgery, respectively [34, 35]. In the present study, the daily administration of tamoxifen in a curative approach mimics this medical application. Results showed that tamoxifen (3.3 mg/kgBW) inhibited tumor growth. Indeed, tamoxifen was reported to induce the apoptosis of cancer cells through the increase in the activity of protein phosphatase 2A (a tumor suppressor protein) and the inhibition of the expression of the cancerous inhibitor of protein phosphatase 2A and phospho-Akt (an oncogenic protein) [47]. Tumor growth inhibition was also observed with the aqueous extract of *D. edulis* leaves. This suggests that *D. edulis* would have used the same signaling pathway as that of tamoxifen to inhibit tumor growth.

Results also showed that tamoxifen decreased cholesterol and increased estradiol levels in the serum of animals. The decrease in serum levels of cholesterol was associated with a significant reduction of this parameter in breast tumors. These effects of tamoxifen on serum and tumor levels of cholesterol are in agreement with the literature data reporting its hypocholesterolemic effect [18]. In addition, the increase in ovary levels of estradiol suggests that tamoxifen would have stimulated the ovarian production of estradiol. Furthermore, the literature reports that tamoxifen is able to stimulate the production of estradiol in nongonadal tissues [18] by stimulating aromatase activity, which is an enzyme responsible for the conversion of androgens to estrogens [48]. Therefore, the gonadal production of estradiol coupled with the peripheral production of this hormone may be responsible for the increase in serum levels of estradiol in animals treated with tamoxifen. However, the increase in serum levels of estradiol was associated with decreased levels of this hormone in the mammary glands and breast tumors of tamoxifen-treated animals. These results are in agreement with the observation of Thomson et al. [49] who reported that tamoxifen antagonizes estrogen receptors in breast tissue as estrogen was reported to stimulate its biosynthesis in its targets tissues [18, 50]. This may justify, at least in part, the decrease in the density of cancer cells in breast tumors as well as the size of breast tumors and that of the mammary alveoli following treatment with tamoxifen.

The aqueous extract of *D. edulis* leaves induced a significant increase of cholesterol in the mammary gland and a significant decrease of this lipid in serum and breast tumors. The hypocholesterolemic effect of the aqueous extract of *D. edulis* leaves in breast tumors was associated with a significant decrease in tumor estradiol levels, indicating the ability of this extract to inhibit the synthesis of this hormone in this tissue. In the mammary gland, the increased levels of cholesterol induced by the aqueous extract of *D. edulis* leaves were rather associated with decreased estradiol levels in this tissue. These results support the aforementioned ability of *D. edulis* to inhibit estradiol synthesis, despite its ability to promote the production of the precursor of this hormone in the mammary gland, by a mechanism which remains to be elucidated. The decrease in tumor and mammary gland levels of estradiol could account for the reduction of alveoli size as well as the atrophy of breast lobules, the decrease in the invasion and density of cancer cells in breast tumors, and the decrease in the volume and weight of tumors. These effects could be attributed to the presence of flavonoids and flavonols (phytoestrogens) in the aqueous extract of *D. edulis* leaves [4]. The so-called phytoestrogens were reported to be able to compete with endogenous estrogens for binding estrogen receptors and to inhibit aromatase activity [51]. As recently suggested by Gul et al. [44], phytoestrogenic molecules are capable of inhibiting estrogen activity (antagonistic effect against ER) or estrogen synthesis (inhibition of aromatase synthesis). The nonsignificant increase in serum levels of estradiol observed at the dose of 100 mg/kg indicates the ability of *D. edulis* to act in a tissue-specific way and to antagonize estrogen receptors in the mammary glands and breast tumors, thus reducing estradiol levels in these tissues, since estradiol is known to stimulate its biosynthesis in estrogen-sensitive tissues [50].

Estrogens are also known for their ability to promote the synthesis of reactive oxygen species (ROS) that cause oxidative stress [52]. The implication of ROS in cancer genesis is widely documented [53–55]. These authors reported that ROS induced DNA mutation and the activation of oncogene, thus promoting cancer genesis [53–55]. According to Visagie et al. [55], in the case of elevated level of oxidative stress, ROS induce mitochondrial membrane damage, decrease metabolic activity, and induce G_2_/M arrest, DNA double-stranded breaks, micronuclei, and apoptosis. In addition, oxidative stress is known to be stimulated by the action of some anticancer and antiproliferative agents [18, 55, 56]. In agreement with these literature data, our results show that tamoxifen increased tumor levels of malondialdehyde (MDA), a lipid peroxidation subproduct and an indicator of oxidative stress [57]. The increase of MDA levels was associated with the increase of antioxidant enzyme (catalase and SOD) activity. As Mvondo et al. [18] recently observed, some antiproliferative agents increase both MDA levels and antioxidant enzymes. In the same line, Rojas-Armas et al. [41] reported that the *in vitro* anticancer effect of essential oil of *Cymbopogon citratus* was attributed to its ability of increasing intracellular ROS and altering the potential of mitochondrial membrane, leading to cell apoptosis [41]. In addition, antioxidant enzymes would protect normal cells from the carcinogenic action of ROS induced by anticancer therapies for the destruction of cancer cells. By increasing the activity of antioxidant enzymes, tamoxifen would in parallel induce a cytoprotective effect on normal cells against the carcinogenic action of ROS, as several authors reported the ability of antioxidant enzymes to protect cells against the action of ROS [53, 54, 58]. The increased levels of antioxidant enzymes could also be the result of a protective mechanism developed by cancer cells against damage caused by ROS. Indeed, the literature reports that cancer cells are able to secrete large amounts of antioxidant molecules in order to protect themselves from damage caused by ROS [59]. This mechanism of adaptation of cancer cells to oxidative stress promotes their survival and development [59] and could be responsible for the observed increase, although slower, in the volume of breast tumors over the weeks. The aforementioned adaptive mechanism has also been implicated in the resistance of cancer cells to anticancer treatments [59].

The increase levels of MDA in the tumors of animals treated with tamoxifen were inversely correlated to the decrease of total protein levels. Proteins are constituent of cell membrane. The literature reports that, in the case of estrogen-sensitive cancers such as breast cancer, an increase of total protein levels is observed due to the anabolic and mitogenic effects of estrogens [60]. According to Thomson et al. [49], tamoxifen acts by inhibiting the effect of estrogen in the mammary gland. In this organ, tamoxifen is reported to exert its effects through mechanisms such as inhibition of Akt phosphorylation and nuclear translocation of NrF2 [17, 47]. Thus, the decrease in total protein levels observed in the present work could result from the inhibition of the anabolic and mitogenic effects of estrogens by tamoxifen, since this selective estrogen receptor modulator has the ability to compete with estrogen for binding to estrogen receptors, thus inhibiting the proliferative effect of this hormone. In addition, the decrease in total protein levels confirms, at least in part, the catabolism of tumor tissue which would probably be stimulated by tamoxifen and thus justify the decrease in the volume and the weight of breast tumors in these animals.

The aqueous extract of *D. edulis* leaves induced a tamoxifen-like effect as it increased the levels of MDA and antioxidant enzymes. The anticancer effect of this extract would be mediated by the increase of ROS production (indicated by elevated levels of MDA), responsible for cell damage [41, 55] and the inhibition of Akt pathway (indicated by elevated levels of antioxidant enzymes), an oncogene protein, where the activation stimulated by ROS is known to inhibit the production of antioxidant enzymes and cell apoptosis, promoting tumor cell survival [53, 54]. However, unlike tamoxifen, the aqueous extract of *D. edulis* leaves did not alter total protein levels in breast tumors, which remained similar to those of the negative control group (DMBA + DW). These elevated levels of total proteins are in contradiction with a real process of cell apoptosis and can justify the observed moderate decrease in the volume of breast tumors. This could be due to the protection system developed by cancer cells *via* the production of antioxidant enzymes [59]. Thus, like tamoxifen, the aqueous extract of *D. edulis* leaves just managed to inhibit the growth of breast tumors by a mechanism which remains to be elucidated.

## 5. Conclusion

This study aimed at evaluating the effects of the aqueous extract of *D. edulis* leaves in animals with already developed breast cancer. It emerges from this study that when cancer occurs, the plant extract manages to inhibit tumor growth. The aqueous extract of *D. edulis* leaves could be therefore considered as a potential alternative for the neoadjuvant treatment of estrogen-dependent breast cancer.

## Figures and Tables

**Table 1:** Effects of a 21-day treatment on breast tumor volume.

Tumor volume (cm^3^)			
DMBA + DW	DMBA + TAM	DMBA + AE 25	DMBA + AE 100
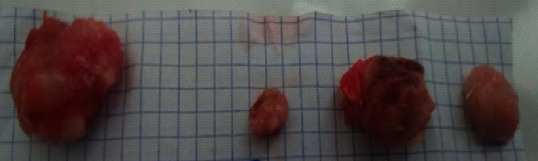			
3.98 ± 0.79	2.40 ± 0.34^#^	3.56 ± 0.71	3.15 ± 0.63

DMBA: 7,12-dimethylbenz[a]anthracene; DW: distilled water; TAM: tamoxifen; AE: aqueous extract of *D. edulis* leaves. Data are expressed as mean ± SEM (*n* = 5). ^#^*p* < 0.05 and ^###^*p* < 0.001 as compared to DMBA + DW.

**Table 2:** Percentage inhibition of tumor growth following treatments.

Tumor volume in cm^3^ (percentage inhibition of tumor growth)				
	Week 0	Week 1	Week 2	Week 3
DMBA + DW	0.72 ± 0.16	2.61 ± 0.58	3.49 ± 0.78	3.98 ± 0.79
DMBA + TAM	0.47 ± 0.12	1.04 ± 0.23^*∗∗∗*^ (60%)	1.75 ± 0.39^*∗∗∗*^ (50%)	2.40 ± 0.34^*∗*^(38%)
DMBA + AE 25	0.46 ± 0.1	2.11 ± 0.47 (19%)	2.63 ± 0.58^*∗∗*^ (25%)	3.56 ± 0.71 (11%)
DMBA + AE 100	0.39 ± 0.1	1.75 ± 0.39^*∗∗*^ (33%)	2.04 ± 0.54^*∗∗∗*^ (42%)	3.15 ± 0.63 (21%)

DMBA: 7,12-dimethylbenz[a]anthracene; DW: distilled water; TAM: tamoxifen; AE: aqueous extract of *D. edulis* leaves. Data are expressed as mean ± SEM (*n* = 5); ^*∗*^*p* < 0.05, ^*∗∗*^*p* < 0.01, and ^*∗∗∗*^*p* < 0.001 as compared to DMBA + DW.

**Table 3:** Effects of treatment on oxidative stress-related parameters in breast tumors.

Groups	MDA (*μ*M/1000 mg of tissue)	Catalase (*μ*mol/mg of total proteins)	SOD Unit of SOD/ml/mg of tissue	Total proteins (mg/dl)
DMBA + DW	1.21 ± 0.27	0.36 ± 0.1	2.88 ± 0.64	1.49 ± 0.33
DMBA + TAM	1.63 ± 0.36	0.94 ± 0.21^###^	4.27 ± 0.95^###^	1.07 ± 0.24^###^
DMBA + AE 25	1.77 ± 0.41	0.7 ± 0.15^##^	3.44 ± 0.77	1.64 ± 0.36
DMBA + AE 100	1.84 ± 0.39^#^	0.84 ± 0.19^###^	3.89 ± 0.87^##^	1.42 ± 0.31

DMBA: 7,12-dimethylbenz[a]anthracene; DW: distilled water; TAM: tamoxifen; AE: aqueous extract of *D. edulis* leaves. Data are expressed as mean ± SEM (*n* = 5). Histomorphology of breast tumors.

## Data Availability

The data used to support the findings of this study are available from the corresponding author upon request.
